# Highly Efficient and Diastereoselective Synthesis of New Pyrazolylpyrrolizine and Pyrazolylpyrrolidine Derivates by a Three-Component Domino Process

**DOI:** 10.3390/molecules19044284

**Published:** 2014-04-04

**Authors:** Jairo Quiroga, Jaime Gálvez, Rodrigo Abonia, Braulio Insuasty, Alejandro Ortíz, Justo Cobo, Manuel Nogueras

**Affiliations:** 1 Heterocyclic Compounds Research Group, Department of Chemistry, Universidad del Valle, Cali 760032, Colombia; E-Mails: jaimegalvez.n@gmail.com (J.G.); rodrigo.abonia@correounivalle.edu.co (R.A.); braulio.insuasty@correounivalle.edu.co (B.I.); alejandro.ortiz@correounivalle.edu.co (A.O.); 2 Department of Inorganic and Organic Chemistry, Universidad de Jaén, Jaén 23071, Spain; E-Mails: jcobo@ujaen.es (J.C.); mmontiel@ujaen.es (M.N.)

**Keywords:** dipyrrolo [3,4-*a*:3',4'-*f*]pyrrolizines, pyrrolo[3,4-*c*]pirroles, 1,3-dipolar cycloadditions, multicomponent reactions, domino reactions

## Abstract

Diastereoselective reactions between 4-formylpyrazoles, *N*-substituted maleimides and glycine derivates led to new series of pyrazolyldipyrrolo [3,4-*a*:3',4'-*f*]pyrrolizines and pyrazolylpyrrolo[3,4-*c*]pyrroles in good yields. The reactions proceeded by a domino process through azomethine ylides formed *in situ* via a 1,3-dipolar cycloaddition reaction.

## 1. Introduction

Multicomponent reactions (MCRs) are one of the most powerful tools in organic synthesis allowing the formation of several bonds in one step to obtain products with high structural diversity and/or molecular complexity [1,2,3]. Furthermore, the development of fast, selective and environmentally friendly MCRs involving domino processes with step and atom economy are of great importance for medicinal and synthetic chemistry [4,5,6].

The dipolar cycloaddition reaction is a known and widely studied method in organic synthesis to obtain pyrrolidine derivatives from the reaction of azomethine ylides, generated *in situ*, and electron-deficient olefins [7,8,9]. These five-membered ring systems belong to an important class of aza-compounds with multiple applications, for example, in the development of bioactive molecules, organocatalysts, new materials and as scaffolds in total organic synthesis [10,11,12,13].

**Figure 1:**
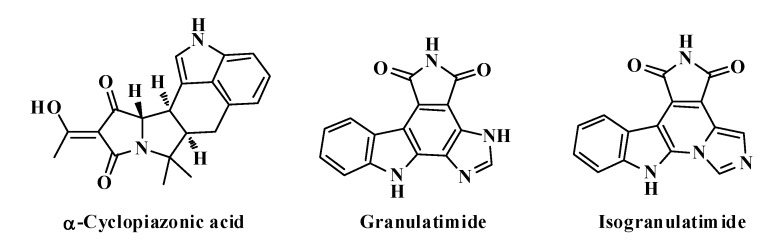
Pyrrolidine systems present in natural alkaloids.

Some interesting fused pyrrolidine systems as cyclopiazonic acid, granulatimide and isogranulatimide (Figure 1) are natural alkaloids that displayed important activities as Chk1 inhibitors and antiplasmodial agents [14,15]. Pyrrolizines are alkaloids generally isolated from plants, insects, bacteria or fungi [16,17] that have exhibited important antiproliferative activities [18,19]. In the same way, the pyrrolo[3,4-*c*]pyrroles have been widely applied in a variety of fields such as materials sciences, pharmaceuticals and agrochemistry [20,21,22].

**Scheme 1:**
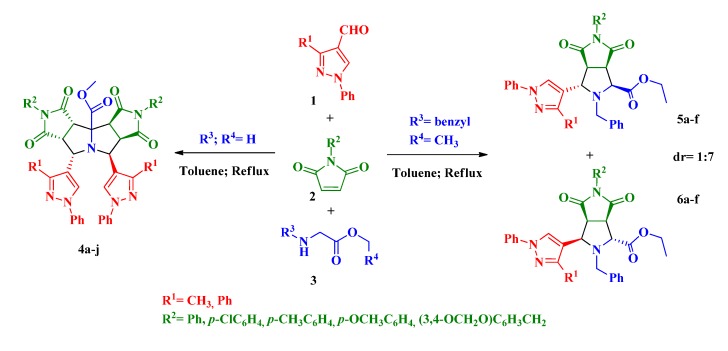
Proposed synthesis of new pyrazolylpyrrolopyrrolizine derivates **4** and pyrazolylpyrrolopyrrolidine derivates **5** and **6**.

Pyrazole is another five-membered ring with many applications in chemistry, especially as pharmaceuticals, pesticides and lubricants [23,24,25,26,27]. In connection with our current studies on the development of new, selective, and environmentally friendly methodologies for the synthesis of fused heterocycles [28,29,30,31], herein we report a procedure for the preparation of the scarcely studied pyrazolylpyrrolizine derivates **4** and pyrazolylpyrrolidine derivatives **5** and **6 ** where three moieties of known importance (*i.e*., pyrazole, pyrrolidine, pyrrolizine) are incorporated into a single structure. The new compounds were obtained in good yields and high diastereoselectivity by a catalyst-free three-component domino reaction between formylpyrazoles **1**, *N*-arylmaleimides **2** and glycine-derived esters **3** (Scheme 1).

## 2. Results and Discussion

### 2.1. Synthesis of Pyrazolyldipyrrolo[3,4-a:3',4'-f]Pyrrolizines from Glycine Methyl Ester

**Scheme 2:**
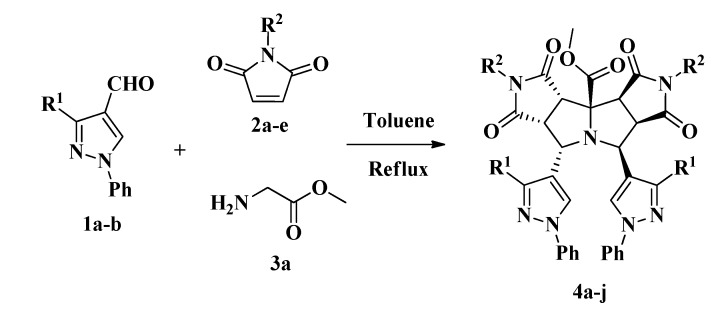
Three-component synthesis of pyrazolyldipyrrolo[3,4-*a*:3',4'-*f*]pyrrolizines **4a**-**j**.

To the best of our knowledge, not many pyrrolo[3,4-*a*:3',4'-*f*]pyrrolizine derivatives have been synthesized and few of them via 1,3-dipolar reactions [32,33,34]. Recently Zhang and coworkers reported the synthesis of pyrrolopyrrolizines from 2-furanyl and 2-thiophenylpyrrolizines by a multicomponent reaction under microwave irradiation using hetarylcarbaldehydes [33]. Similarly, our synthesis (Scheme 2) was carried out by a three-component combinatorial methodology between formylpyrazoles **1**, *N*-substituted-maleimides **2** and glycine methyl ester **3** in refluxing toluene affording the products **4** via a double cycloaddition reaction (Table 1).

**Table 1:** Synthesis of diverse pyrazolyldipyrrolo[3,4-*a*:3',4'-*f*]pyrrolizines **4a**–**j**.

Entry	R^1^	R^2^	Yield (%)
**4a**	-CH_3_	C_6_H_5_	96
**4b**	-CH_3_	*p*-ClC_6_H_4_	73
**4c**	-CH_3_	*p*-CH_3_C_6_H_4_	84
**4d**	-CH_3_	*p*-CH_3_OC_6_H_4_	90
**4e**	-CH_3_	(3,4-OCH_2_O)C_6_H_3_CH_2_	78
**4f**	C_6_H_5_	C_6_H_5_	81
**4g**	C_6_H_5_	*p*-ClC_6_H_4_	75
**4h**	C_6_H_5_	*p*-CH_3_C_6_H_4_	93
**4i**	C_6_H_5_	*p*-CH_3_OC_6_H_4_	94
**4j**	C_6_H_5_	(3,4-OCH_2_O)C_6_H_3_CH_2_	75

**Scheme 3:**
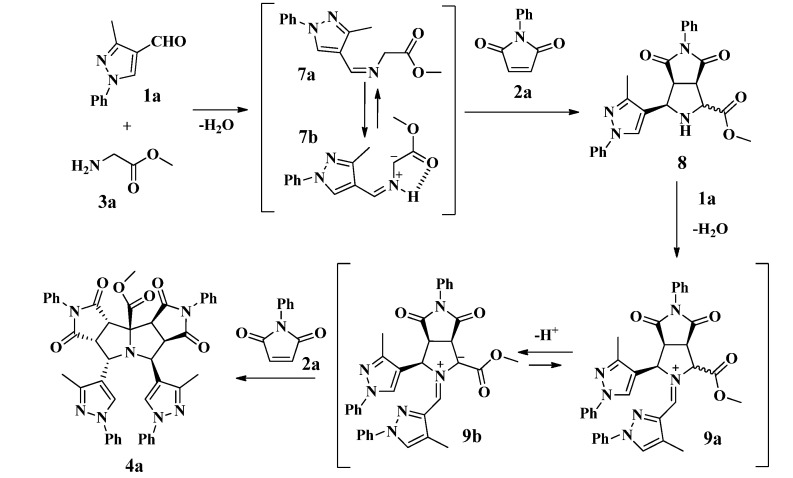
Proposed mechanism for the synthesis of pyrazolyldipyrrolo[3,4-*a*:3',4'-*f*] pyrrolizine **4a**.

The reaction consists of a domino process as shown in Scheme 3. We propose that initially the condensation of the glycine derivative **3a** with the formylpyrazole **1a** produced the imine **7a**; subsequently, a 1,2-proton shift in imine **7a** should afford the azomethine ylide **7b**, which in turn should be trapped by the maleimide **2a** to generate the intermediate pyrrolopyrrolidine derivative **8 ** (Scheme 3) [28].

A second condensation should take place between the pyrrolidine intermediate **8** and the formylpyrazole **1a** affording the iminium ion **9a**, which should generate the 1,3-dipolar azomethine ylide **9b** by deprotonation of its acidic proton adjacent to the carbomethoxy group. Finally, the tetracyclic product **4a** would be formed by the diastereoselective 1,3-cycloaddition of a second molecule of maleimide **2a** to the ylide **9b**. During the process, the formation of the azomethine ylides **7b** and **9b** should be favored by the acidity of the α-protons in the moieties **7a** and **9a** and the *in situ* stability of these species due to an intramolecular H-bond and π-conjugation [8,35,36,37].

**Figure 2:**
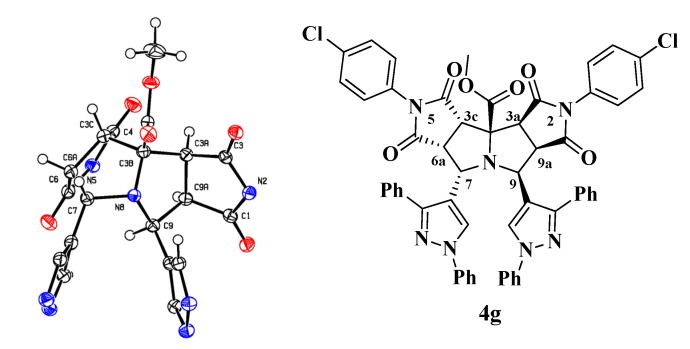
ORTEP drawing of the compound **4g** with 50% probability elipsoids. In the ORTEP view of the tetracyclic scaffold, the aryl pendant groups have been removed for the sake of clarity.

The structural elucidation of the new compounds **4a**–**j** was made by analysis of the corresponding NMR, infrared and mass spectrometry data. The ^1^H-NMR spectra of compounds **4a**–**j** show six aliphatic signals corresponding to the protons on the stereogenic centers of the dipyrrolopyrrolizine framework. Two of them appear as triplets corresponding to the H-6a and H-9a protons, and the remaining four appear as doublets assigned to the H-3a, H-3c, H-7 and H-9 protons. Crystals of compound **4g** suitable for single-crystal X-ray diffraction were obtained by slow evaporation from a DMF:EtOH (1:1), thus solution confirming the structure and stereochemistry of the racemic compounds **4a**–**j** (Figure 2) [38].

### 2.2. Synthesis of Pyrazolylpyrrolo[3,4-c]Pyrroles from N-benzylglicine Ethyl Ester

**Scheme 4:**
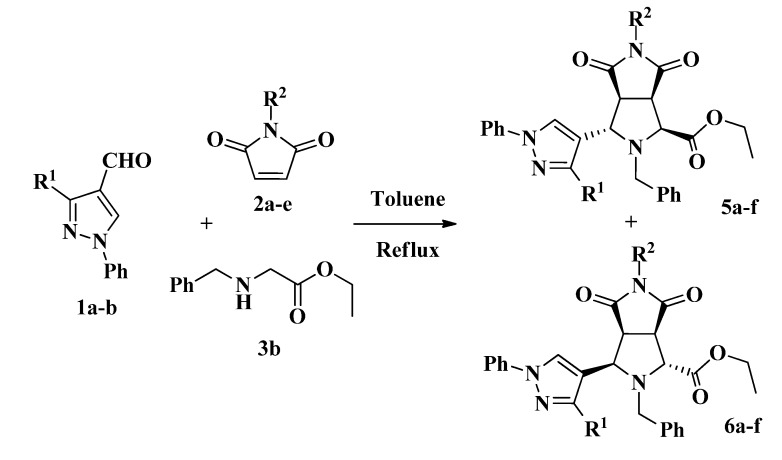
Three-component synthesis of pyrazolylpyrrolo[3,4-*c*]pyrroles **5** and **6**.

On the other hand, it is known that a similar three-component reaction using α-amino acids can be stopped at the pyrazolyl-pyrrolo[3,4-*c*]pyrroles **8** if the second cycloaddition is blocked by replacing the proton on the α-carbon atom by an alkyl group [28,29]. Thus, in order to preclude the formation of compounds type **4**, we performed the reaction with *N*-benzylglycine ethyl ester **3b** instead of **3a**, along with the formylpyrazoles **1a**–**b** and maleimides **2a**–**e** (Scheme 4). As anticipated, the second amino condensation on the pyrrolidine nitrogen was blocked and the new compounds **5a**–**f** and **6a**–**f** were obtained in a diastereoselective manner with good yields (Table 2).

**Table 2:** Synthesis of diverse pyrazolylpyrrolo[3,4-*c*]pyrroles **5** and **6**.

Entry	R^1^	R^2^	d:r ^a^ (5:6)	Yield (%) 5 + 6
1	-CH_3_	C_6_H_5_	1:7	95
2	-CH_3_	*p*-ClC_6_H_4_	1:7	82
3	-CH_3_	*p*-CH_3_OC_6_H_4_	1:7	96
4	C_6_H_5_	C_6_H_5_	1:7	75
5	C_6_H_5_	*p*-ClC_6_H_4_	1:7	72
6	C_6_H_5_	*p*-CH_3_OC_6_H_4_	1:7	90

^a^ Determinated by NMR.

**Scheme 5:**
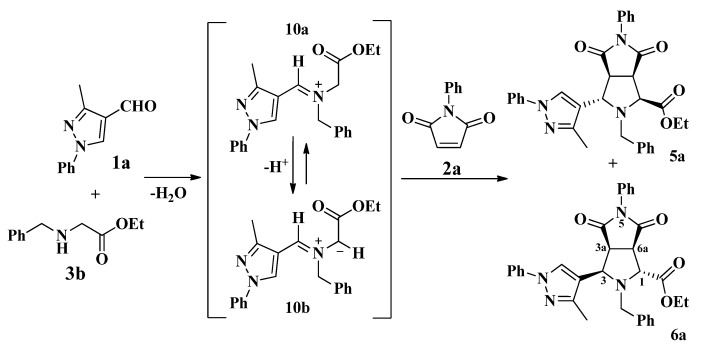
Proposed mechanism for the synthesis of pyrazolylpyrrolo[3,4-*c*]pyrroles **5a** and **6a**.

In this approach, the first step is a condensation between the formylpyrazole **1a** and the *N*-benzyl glycine ethyl ester **3b** affording the iminium ion **10a**, which is subsequently deprotonated giving azomethine ylide **10b** (Scheme 5). Then, the 1,3-dipolar cycloaddition of **10b** with the *N*-phenyl maleimide **2a** afforded the diastereomers **5a** and **6a**. In all cases, the reaction showed good diastereoselectivity toward isomers **6**, in which repulsive interactions between the ester group on the C-1 and C-6 carbonyl group are avoided due to the *trans* configuration between them as shown in **6a** (Scheme 5) [17].

According to the ^1^H-NMR analysis of compounds **6** the H-1 proton on the stereogenic center appears as a singlet due to the dihedral angle (aprox. 90°) with the H-6a proton indicating a *trans* configuration. On the other hand, both the H-3 and H-6a protons appear as doublets with coupling constants about 9.0 Hz corresponding to a *cis* configuration with the H-3a proton, which in turn appears as a double doublet. Meantime, in compounds **5** the H-1 proton is observed as a doublet with a coupling constant *J *≈ 9.0 Hz because of its *cis* configuration with respect to the H-6a proton, while the H-3 proton appears as a doublet with a smaller coupling constant (*J *≈ 5.5 Hz) due to a *trans* configuration with respect to the H-3a proton [39].

### 2.3. Theoretical Calculations

**Figure 3:**
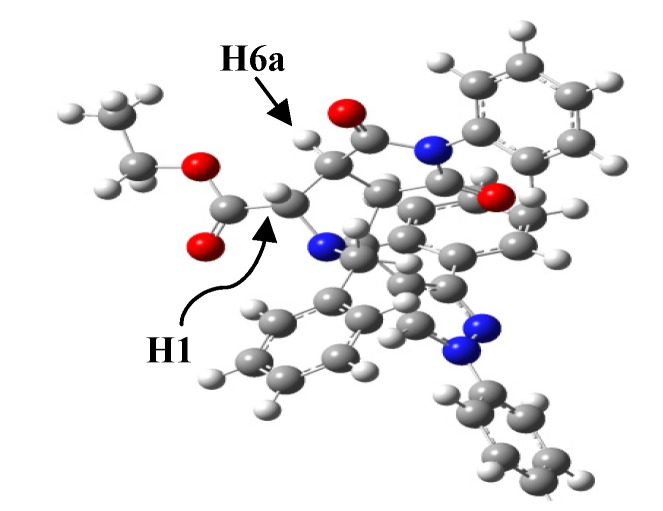
Minimum-energy configuration calculated for the compound **6d** at the B3LYP/6-311G++ level. Dihedral Angle (1H-C-C-6aH) = −94.41°.

To confirm our findings about the stereochemistry of the reactions as well as the obtained compounds **4**–**6**, theoretical calculations were carried out with the DFT approach using the B.01 revision of the Gaussian 09 program package [40]. DFT calculations were performed using Becke’s three-parameter B3LYP exchange-correlation functional and the 6-311G++ basis set. The geometry of compound **6d** was theoretically optimized and the most stable configuration is depicted in Figure 3. Although the found energy values for the *cis* and *trans* configurations of the stereoisomers **5d** and **6d** are very close, the *trans* configuration for **6d** is slightly favored by 7.82696 × 10^−17^ Kcal over the *cis* form for **5d**. This finding is in agreement with the Karplus theory [41] and with our experimental ^1^H-NMR measurements, since the dihedral angle 1H-C-C-6aH for the stereoisomer **6d** is −94.41° and therefore in its ^1^H-NMR spectrum the coupling constant between the H-1 and H-6a protons has a value *J*_3_~0.0 Hz.

**Figure 4:**
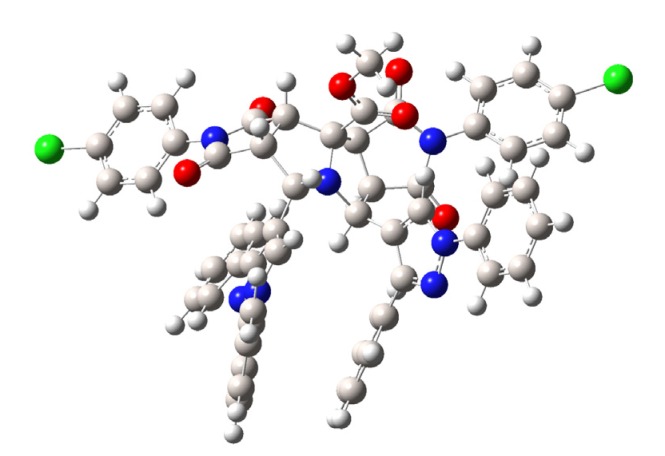
Minimum-energy configuration calculated for compound **4g** at the B3LYP/6-311G++ level.

In order to verify the accuracy of the theoretical method, the geometry of compound **4g** was theoretically optimized and the most stable configuration is depicted in Figure 4. Geometrical parameters such as bond length and molecular angles were calculated. We observed that the calculated parameters were very close to the experimental values, measured by X-ray diffraction. Some of these geometrical stocks are listed in Table 3.

**Table 3:** Geometrical parameters of compound **4g**; X-ray data is compared with theoretical calculated parameters.

Bond	X-Ray Length (Å)	Calculated Length (Å)	Angle Atoms	X-Ray Angle (°)	Calculated Angle (°)
**C1-O1**	1.210(2)	1.213	O1-C1-N2	124.61(17)	125.02
**C1-N2**	1.393(2)	1.398	O1-C1-C8a	128.00(16)	127.64
**C1-C8a**	1.508(3)	1.512	N2-C1-C8a	107.36(14)	110.73
**N2-C3**	1.399(2)	1.409	C1-N2-C3	112.48(15)	114.34
**C3-O3**	1.209(2)	1.211	C1-N2-C21	122.84(14)	120.21
**C3-C3a**	1.529(3)	1.534	C3-N2-C21	124.47(15)	124.88
**C3c-C4**	1.507(3)	1.511	O3-C3-N2	124.53(17)	123.00

## 3. Experimental

### 3.1. General Information

The pyrazole-4-carbaldehydes **1a**–**b** and the glycine ester derivates **3a**–**b** (analytical reagent grade) were purchased from Aldrich (St. Louis, Missouri, United State), Fluka (St. Louis, MO, USA) and Merck (Darmstadt, Alemania) and were used without further purification. The maleimides **2a**–**e** were obtained according to the already reported procedure [42]. Solvents and other chemicals commercially available were used as shipped. Silica gel aluminium plates (Merck 60 F_254_) were used for analytical TLC. Melting points were taken on Stuart SMP10 Melting point apparatus and are uncorrected. IR spectra were recorded on a Shimadzu FTIR 8400 spectrophotometer using KBr disks or CH_2_Cl_2_ as solvent. ^1^H- and ^13^C-NMR were recorded on a Bruker Avance 400 spectrometer operating at 400 and 100 MHz respectively, using CDCl_3_ as solvent. Mass spectra were obtained on a Shimadzu GCMS-QP 2010 spectrometer (equipped with a direct inlet probe) operating at 70 eV and with a Bruker Esquire 6000 spectrometer equipped with an electrospray ionization source and an ion-trap detector. Microanalyses were performed on a LECO CHNS-900 elemental analyzer and the values are within ± 0.4% of theoretical values.

### 3.2. Synthesis and Characterization Data for Pyrazolyldipyrrolo[3,4-a:3',4'-f]Pyrrolizines ***4a–j***

#### General Synthetic Procedure

To a 25.0 mL round bottom flask equipped with a magnetic stirring bar and a reflux condenser were added pyrazole-4-carboxaldehyde **1a**–**b** (0.2 mmol), *N*-substituted-maleimide **2a**–**e** (0.2 mmol), glycine methyl ester **3a** (0.2 mmol) and toluene (8 mL). The mixture was heated under reflux for 8–10 h. The reaction mixture was cooled to ambient temperature and the resulting precipitate was collected by filtration and washed with hexane-toluene (1:1) to obtain the pure compounds. In some cases, the solid was recrystallized from a mixture ethanol-DMF (1:1) to obtain the pure compound **4**.

*Methyl 7,9-bis(3-methyl-1-phenyl-1H-pyrazol-4-yl)-1,3,4,6-tetraoxo-2,5-diphenyldodecahydro-1H-dipyrrolo[3,4-a:3',4'-f]pyrrolizine-3b-carboxylate* (**4a**). Beige solid. Yield: 96%; m.p.: 182–184 °C. IR (KBr): ν 3473, 2954, 1753, 1715 cm^−1^. ^1^H-NMR (400 MHz, CDCl_3_) δ ppm: 2.01 (s, 3H), 2.26 (s, 3H), 3.51 (dd, *J * = 8.0 Hz; *J * = 8.3 Hz, 1H), 3.74 (t, *J * = 8.0 Hz, 1H), 3.99 (s, 3H), 4.29 (d, *J * = 8.3 Hz, 1H), 4.46 (d, *J * = 10.5 Hz, 1H), 4.64 (d, *J * = 8.3 Hz, 1H), 4.91 (d, *J * = 7.8 Hz, 1H), 6.81 (d, *J * = 8.8 Hz, 2H), 7.18 (d, *J * = 8.5 Hz, 2H), 7.20–7.26 (m, 2H), 7.28–7.39 (m, 12H), 7.53 (s, 1H), 7.57 (d, *J * = 8.8 Hz, 2H), 7.98 (s, 1H). ^13^C-NMR (100 MHz, CDCl_3_) δ ppm: 11.5 (CH_3_), 11.9 (CH_3_), 48.2 (CH), 48.9 (CH), 49.5 (CH), 53.2 (CH), 53.5 (OCH_3_), 58.1 (CH), 60.6 (CH), 79.34 (C), 112.2 (C), 118.4 (CH), 118.8 (CH), 119.4 (CH), 120.1 (C), 125.9 (CH), 126.3 (CH), 126.4 (CH), 126.8 (CH), 126.9 (CH), 127.3 (CH), 129.1 (CH), 129.2 (CH), 129.3 (CH), 129.40 (CH), 130.0 (CH), 134.3 (C), 135.4 (C), 139.6 (C), 139.7 (C), 146.6 (C), 150.7 (C), 170.2 (C), 171.7 (C), 173.2 (C), 174.7 (C), 175.7 (C). MS (EI, 70 eV) *m/z* (%): 771 (M^+^, 2), 712 (13), 598 (100), 539 (70), 268 (27), 211 (32), 171 (75). Elemental Analyses calcd. for C_45_H_37_N_7_O_6_.2H_2_O: C: 66.90, H: 5.12, N: 12.14. Found: C: 66.75, H: 5.47, N: 12.07.

*Methyl 2,5-bis(4-chlorophenyl)-7,9-bis(3-methyl-1-phenyl-1H-pyrazol-4-yl)-1,3,4,6 tetraoxododeca-hydro-1H-dipyrrolo[3,4-a:3',4'-f]pyrrolizine-3b-carboxylate* (**4b**). Pale yellow solid. Yield: 73%; m.p.: 170–172 °C. IR (KBr): ν 3479, 2953, 1716 cm^−1^. ^1^H-NMR (400 MHz, CDCl_3_) δ ppm: 2.03 (s, 3H), 2.29 (s, 3H), 3.53 (dd, *J * = 10.7, 8.2, 1H), 3.76 (t, *J * = 8.0 Hz, 1H), 4.02 (s, 3H), 4.31 (d, *J * = 8.3 Hz, 1H), 4.48 (d, *J * = 10.5 Hz, 1H), 4.66 (d, *J * = 8.3 Hz, 1H), 4.94 (d, *J * = 7.8 Hz, 1H), 6.83 (d, *J * = 8.8 Hz, 2H), 7.21 (d, *J * = 8.5 Hz, 2H), 7.26 (m, 2H), 7.37 (m, 10H), 7.55 (s, 1H), 7.60 (d, *J * = 8.8 Hz, 2H), 8.00 (s, 1H). ^13^C-NMR (100 MHz, CDCl_3_) δ ppm: 11.5 (CH_3_), 11.9 (CH_3_), 48.2 (CH), 48.9 (CH), 49.5 (CH), 53.2 (CH); 53.5 (OCH_3_), 58.1 (CH), 60.6 (CH), 79.3 (C), 112.2 (C) 118.8 (CH), 119.4 (CH), 120.1 (C), 125.9 (CH), 126.3 (CH), 126.4 (CH), 126.8 (CH), 126.9 (CH), 127.3 (CH), 129.1 (CH), 129.2 (CH), 129.3 (CH), 129.4 (C), 129.7 (C), 130.0 (CH), 134.3 (C), 135.4 (C), 139.6 (C), 139.7 (C), 146.6 (C), 150.7 (C), 170.2 (C), 171.7 (C), 173.2 (C), 174.7 (C), 175.7 (C). MS (EI, 70 eV) *m/z* (%): 839 (M^+^, 2), 780 (18), 632 (42), 268 (22), 211 (39), 171 (100). Elemental Analyses calcd. for C_45_H_35_Cl_2_N_7_O_6_.H_2_O: C: 62.94, H: 4.34, N: 11.42. Found: C: 63.32, H: 4.36, N: 11.34.

*Methyl 7,9-bis(3-methyl-1-phenyl-1H-pyrazol-4-yl)-1,3,4,6-tetraoxo-2,5-di-p-tolyldodecahydro-1H-dipyrrolo[3,4-a:3',4'-f]pyrrolizine-3b-carboxylate* (**4c**). Beige solid. Yield: 84%; m.p.: 179–181 °C. IR (KBr): ν 3647, 2952, 1784, 1756, 1696 cm^−1^. ^1^H-NMR (400 MHz, CDCl_3_) δ ppm: 2.02 (s, 3H), 2.25 (s, 3H), 2.27 (s, 3H), 2.46 (s, 3H), 3.56 (dd, *J * = 10.7, 8.2 Hz, 1H), 3.71–3.77 (m, 1H), 4.00 (s, 3H), 4.28 (d, *J * = 8.3 Hz, 1H), 4.51 (d, *J * = 10.8 Hz, 1H), 4.66 (d, *J * = 8.3 Hz, 1H), 4.93 (d, *J * = 8.0 Hz, 1H), 6.75 (d, *J * = 8.3 Hz, 2H), 7.03 (d, *J * = 7.5 Hz, 2H), 7.21–7.25 (m, 4H), 7.33–7.39 (m, 5H), 7.39–7.44 (m, 5H), 7.58 (s, 1H), 8.04 (s, 1H). ^13^C-NMR (100 MHz, CDCl_3_) δ ppm: 11.5 (CH_3_), 11.9 (CH_3_), 21.0 (CH_3_), 21.3 (CH_3_), 48.2 (CH), 48.8 (CH), 49.6 (CH), 53.3 (CH), 53.4 (OCH_3_), 58.1 (CH), 60.5 (CH), 79.3 (C), 112.5 (C), 118.4 (CH), 118.9 (CH), 119.4 (CH), 120.3 (C), 125.4 (CH), 125.5 (CH), 125.7 (CH), 126.1 (CH), 126.3 (CH), 127.5 (CH), 128.4 (C), 128.6 (C), 129.2 (CH), 129.6 (CH), 130.4 (CH), 138.7 (C), 139.6 (C), 139.7 (C), 139.8 (C), 146.7 (C), 150.7 (C), 170.4 (C), 172.2 (C), 173.7 (C), 175.1 (C), 176.2 (C). MS (EI, 70 eV) *m/z* (%): 799 (M^+^, 2), 740 (8), 612 (100), 553 (18), 257 (60), 197 (75). Elemental Analyses calcd. for C_47_H_41_N_7_O_6_: C: 70.57, H: 5.17, N: 12.26. Found: C: 70.87, H: 5.41, N: 11.90.

*Methyl 2,5-bis(4-methoxyphenyl)-7,9-bis(3-methyl-1-phenyl-1H-pyrazol-4-yl)-1,3,4,6-tetraoxododeca-hydro-1H-dipyrrolo[3,4-a:3',4'-f]pyrrolizine-3b-carboxylate* (**4d**). White Solid. Yield: 90%; m.p.: 174–176 °C. IR (KBr): ν 3478, 2955, 1714, 1598 cm^−1^. ^1^H-NMR (400 MHz, CDCl_3_) δ ppm: 2.02 (s, 3H), 2.27 (s, 3H), 3.54 (dd, *J * = 10.7, 8.2 Hz, 1H), 3.68–3.75 (m, 4H), 3.88 (s, 3H), 4.00 (s, 3H), 4.26 (d, *J * = 8.3 Hz, 1H), 4.50 (d, *J * = 10.5 Hz, 1H), 4.65 (d, *J * = 8.0 Hz, 1H), 4.92 (d, *J * = 7.8 Hz, 1H), 6.72 (d, *J * = 9.0 Hz, 2H), 6.75 (d, *J * = 9.0, 2H), 7.10 (d, *J * = 9.0 Hz, 2H), 7.20–7.29 (m, 4H), 7.30–7.44 (m, 8H), 7.57 (s, 1H), 8.05 (s, 1H). ^13^C-NMR (100 MHz, CDCl_3_) δ ppm: 11.5 (CH_3_), 11.9 (CH_3_), 48.2 (CH), 48.8 (CH), 49.6 (CH), 53.3 (CH), 53.4 (OCH_3_), 55.3 (OCH_3_), 55.6 (OCH_3_), 58.1 (CH), 60.5 (CH), 79.2 (C), 112.5 (C), 114.3 (CH), 115.1 (CH), 118.9 (CH), 119.4 (CH), 120.4 (C), 123.7 (C), 123.8 (C), 126.1 (CH), 126.3 (CH), 126.8 (CH), 126.9 (CH), 127.5 (CH), 129.2 (CH), 139.7 (C), 139.8 (C),146.7 (C), 150.7 (C), 159.3 (C), 160.1 (C), 170.4 (C), 172.3 (C), 173.8 (C), 175.2 (C), 176.3 (C). MS (EI, 70 eV) *m/z* (%): 831 (M^+^, 2), 772 (8), 628 (67), 268 (43), 203 (46), 171 (100). Elemental Analyses calcd. for C_47_H_45_N_7_O_10_: C: 65.04, H: 5.23, N: 11.30. Found: C: 65.44, H: 4.90, N: 11.27.

*Methyl 2,5-bis(benzo[d][1,3]dioxol-5-ylmethyl)-7,9-bis(3-methyl-1-phenyl-1H-pyrazol-4-yl)-1,3,4,6-tetraoxododecahydro-1H-dipyrrolo[3,4-a:3',4'-f]pyrrolizine-3b-carboxylate* (**4e**). Beige Solid. Yield: 78%; m.p.: 243–245 °C. IR (KBr): ν 3459, 2898, 1749, 1694, 1598 cm^−1^. ^1^H-NMR (400 MHz, CDCl_3_) δ ppm: 1.56 (s, 3H), 2.11 (s, 3H), 2.91 (dd, *J * = 10.5, 8.5 Hz, 1H), 3.51 (t, *J * = 8.4 Hz, 1H), 3.65 (d, *J * = 10.5 Hz, 1H), 3.95 (s, 3H), 4.22 (d, *J * = 8.3 Hz, 1H), 4.23–4.34 (m, 2H), 4.49 (d, *J * = 8.5 Hz, 1H), 4.63–4.74 (m, 3H), 5.71–5.83 (m, 2H), 5.86–5.96 (m, 2H), 6.47 (d, *J * = 7.0 Hz, 1H), 6.63 (d, *J * = 7.3 Hz, 1H), 6.70 (s, 1H), 6.80 (d, *J * = 7.8 Hz, 1H), 6.87–7.01 (m, 1H), 7.08–7.11 (m, 1H), 7.12 (d, *J * = 1.5 Hz, 1H), 7.15–7.23 (m, 5H), 7.27–7.37 (m, 5H), 7.52 (s, 1H). ^13^C-NMR (100 MHz, CDCl_3_) δ ppm: 11.2 (CH_3_), 11.3 (CH_3_), 42.2 (CH_2_), 42.9 (CH_2_), 48.6 (CH), 48.7 (CH), 48.8 (CH), 52.5 (CH), 53.4 (OCH_3_), 59.7 (CH), 63.6 (CH), 79.3 (C), 101.0 (CH_2_), 101.5 (CH_2_), 108.1 (CH), 108.9 (CH), 109.1 (CH), 109.6 (CH), 118.5 (CH), 119.2 (CH), 122.3 (CH), 122.4 (CH), 123.3 (CH), 125.5 (CH), 125.6 (CH), 126.2 (CH), 128.1 (C), 128.8 (C), 129.0 (CH), 129.2 (CH), 139.6 (C), 139.7 (C), 147.1 (C), 147.5 (C), 148.2 (C), 148.4 (C), 150.2 (C), 170.7 (C), 172.8 (C), 174.7 (C), 175.8 (C), 176.4 (C). **MS** (EI, 70 eV) *m/z* (%): 887 (M^+^, 1), 657 (20), 656 (50), 231 (16), 171 (74), 135 (100). Elemental Analyses calcd. for C_49_H_41_N_7_O_10_.2H_2_O: C: 63.70, H: 4.91, N: 10.61. Found: C: 64.03, H: 5.15, N: 10.82.

*Methyl 7,9-bis(1,3-diphenyl-1H-pyrazol-4-yl)-1,3,4,6-tetraoxo-2,5-diphenyldodecahydro-1H-dipyrrolo- [3,4-a:3',4'-f]pyrrolizine-3b-carboxylate* (**4f**). Yellow Solid. Yield: 81%; m.p.: 284–286 °C. IR (KBr): ν 3062, 2954, 1746, 1712, 1598 cm^−1^. ^1^H-NMR (400 MHz, CDCl_3_-*d*) δ ppm: 3.59 (t, *J * = 8.4 Hz, 1H), 3.78–3.85 (m, 1H), 3.97 (s, 3H), 4.36 (d, *J * = 8.5 Hz, 1H), 4.72 (d, *J * = 10.5 Hz, 1H), 4.82 (d, *J * = 8.3 Hz, 1H), 5.15 (d, *J * = 8.5 Hz, 1H), 6.97 (dd, *J * = 6.5, 2.8 Hz, 2H), 7.02–7.08 (m, 2H), 7.11–7.17 (m, 3H), 7.21–7.25 (m, 5H), 7.28–7.35 (m, 7H), 7.37–7.51 (m, 7H), 7.53–7.59 (m, 3H), 7.83 (s, 1H), 7.91 (d, *J * = 7.3 Hz, 2H). ^13^C-NMR (100 MHz, CDCl_3_) δ ppm: 48.7 (CH), 49.5 (CH), 49.6 (CH), 52.9 (CH), 53.7 (OCH_3_), 59.2 (CH), 60.7 (CH), 80.1 (C), 111.0 (C), 119.3 (CH), 119.8 (CH), 120.3 (C), 125.7 (CH), 126.1 (CH), 126.5 (CH), 126.7 (CH), 127.1 (CH), 127.9 (CH), 128.0 (CH), 128.2 (CH), 128.3 (CH), 128.5 (CH), 128.6 (CH), 128.6 (CH), 129.0 (CH), 129.1 (CH), 129.2 (CH), 129.3 (CH), 129.4 (CH), 129.8 (CH), 131.1 (C), 131.2 (C), 132.3 (C), 132.5 (C), 139.6 (C), 139.6 (C), 150.5 (C), 152.7 (C), 170.1 (C), 172.5 (C), 173.7 (C), 175.3 (C), 176.0 (C). MS (EI, 70 eV) *m/z* (%): 895(M^+^, 1), 836 (6), 722 (71), 298 (23), 273 (27), 233 (100), 173 (42). Elemental Analyses calcd. for C_55_H_45_N_7_O_6_.3H_2_O: C: 69.54, H: 4.99, N: 10.32. Found: C: 69.43, H: 4.94, N: 10.42.

*Methyl 2,5-bis(4-chlorophenyl)-7,9-bis(1,3-diphenyl-1H-pyrazol-4-yl)-1,3,4,6-tetraoxododecahydro-1H-dipyrrolo[3,4-a:3',4'-f]pyrrolizine-3b-carboxylate* (**4g**). White Solid. Yield: 75%; m.p.: 275–277 °C. IR (KBr): ν 3447, 1718, 1598, 1496 cm^−1^. ^1^H-NMR (400 MHz, CDCl_3_) δ ppm: 3.50–365 (m, 1H); 3.72–3.80 (m, 1H); 3.96 (s, 3H); 4.38 (d, *J * = 8.53 Hz, 1H); 4.66 (d, *J * = 10.54 Hz, 1H); 4.79 (d, *J * = 8.28 Hz, 1H); 5.16 (d, *J * = 8.53 Hz, 1H); 6.92–7.03 (m, 4H) 7.12–7.32 (m, 13H), 7.33–7.44 (m, 6H), 7.45–7.55 (m, 4H), 7.75 (s, 1H); 7.89 (d, *J * = 7.78 Hz, 2H). ^13^C-NMR (100 MHz, CDCl_3_) δ ppm: 48.6 (CH); 49.3 (CH); 49.6 (CH); 52.8 (CH); 53.7 (CH_3_); 59.2 (CH); 60.7 (CH); 80.2 (C); 110.8 (C) 119.2 (CH); 119.8 (CH); 120.1 (C); 125.3 (C); 126.6 (CH); 126.8 (CH); 127.0 (CH); 127.4 (CH); 127.9 (CH); 128.0 (CH); 128.2 (CH); 128.4 (CH); 128.6 (CH); 128.7 (CH); 128.9 (C); 129.1 (CH); 129.2 (CH); 129.3 (CH); 129.4 (CH); 129.5 (CH); 129.6 (C); 130.0 (CH); 132.2 (C); 132.5 (C); 134.5 (C); 135.5 (C), 139.5 (C); 139.6 (C); 150.4 (C); 152.7 (C); 169.9 (C); 172.1 (C); 173.3 (C); 175.1 (C). MS (EI, 70 eV) *m/z* (%): 963(M^+^), 756 (31), 523 (10), 298 (18), 273 (16), 233 (100), 207 (30). Elemental Analyses calcd. for C_55_H_39_Cl_2_N_7_O_6_.4H_2_O: C: 63.71, H: 4.57, N: 9.46. Found: C: 63.74, H: 4.42, N: 9.37.

*Methyl 7,9-bis(1,3-diphenyl-1H-pyrazol-4-yl)-1,3,4,6-tetraoxo-2,5-di-p-tolyldodecahydro-1H-dipyrrolo- [3,4-a:3',4'-f]pyrrolizine-3b-carboxylate* (**4h**). White Solid. Yield: 93%; m.p.: 204–206 °C. IR (KBr): ν 3574, 2954, 1747, 1711, 1599 cm^−1^. ^1^H-NMR (400 MHz, CDCl_3_) δ ppm: 2.26 (s, 3H), 2.50 (s, 3H), 3.59 (t, *J * = 8.4 Hz, 1H), 3.80 (dd, *J * = 10.5, 8.3 Hz, 1H), 3.96 (s, 3H), 4.35 (d, *J * = 8.3 Hz, 1H), 4.71 (d, *J * = 10.8 Hz, 1H), 4.80 (d, *J * = 8.3 Hz, 1H), 5.14 (d, *J * = 8.5 Hz, 1H), 6.84 (d, *J * = 8.3 Hz, 2H), 6.93 (d, *J * = 8.0 Hz, 2H), 7.03 (d, *J * = 8.0 Hz, 2H), 7.12–7.18 (m, 2H), 7.18–7.24 (m, 1H), 7.26–7.30 (m, 5H), 7.30–7.44 (m, 10H), 7.44–7.50 (m, 3H), 7.82 (s, 1H), 7.90 (d, *J * = 7.0 Hz, 2H). ^13^C-NMR (100 MHz, CDCl_3_) δ ppm: 21.1 (CH_3_), 21.4 (CH_3_), 48.8 (CH), 49.5 (CH), 49.6 (CH), 52.9 (CH), 53.7 (OCH_3_), 59.1 (CH), 60.7 (CH), 80.1 (C), 100.0 (C), 111.0 (C), 119.3 (CH), 119.8 (CH), 120.4 (C), 125.5 (CH), 126.0 (CH), 126.4 (CH), 126.7 (CH), 127.2 (CH), 127.9 (CH), 128.0 (CH), 128.2 (CH), 128.3 (CH), 128.5 (CH), 128.6 (CH), 128.7 (CH), 129.2 (CH), 129.3 (CH), 129.7 (CH), 130.4 (CH), 132.3 (C), 132.6 (C), 138.6 (C), 138.7 (C), 139.6 (C), 139.7 (C), 139.8 (C), 150.5 (C), 152.7 (C), 170.2 (C), 172.6 (C), 173.8 (C), 175.4 (C), 176.2 (C). MS (EI, 70 eV) *m/z* (%): 923(M^+^), 865 (5), 737 (95), 503 (16), 298 (35), 233 (100), 187 (45). Elemental Analyses calcd. for C_57_H_45_N_7_O_6_.H_2_O: C: 72.67, H: 5.03, N: 10.41. Found: C: 72.99, H: 5.24, N: 10.50.

*Methyl 7,9-bis(1,3-diphenyl-1H-pyrazol-4-yl)-2,5-bis(4-methoxyphenyl)-1,3,4,6-tetraoxododecahydro-1H-dipyrrolo[3,4-a:3',4'-f]pyrrolizine-3b-carboxylate* (**4i**). Beige Solid. Yield: 94%; m.p.: 239–241 °C. IR (KBr): ν 3474, 2955, 1714, 1599 cm^−1^. ^1^H-NMR (400 MHz, CDCl_3_) δ ppm: 3.57 (t, *J* = 8.4 Hz, 1H), 3.70 (s, 3H), 3.76–3.80 (m, 1H), 3.92 (s, 3H), 3.96 (s, 3H), 4.33 (d, *J* = 8.3 Hz, 1H), 4.68 (d, *J* = 10.8 Hz, 1H), 4.79 (d, *J* = 8.3 Hz, 1H), 5.14 (d, *J* = 8.3 Hz, 1H), 6.73 (d, *J* = 9.0 Hz, 2H), 6.88 (d, *J* = 8.8 Hz, 2H), 6.95 (d, *J* = 8.8 Hz, 2H), 7.05 (d, *J* = 8.8 Hz, 2H), 7.12–7.122 (m, 4H), 7.24–7.36 (m, 9H), 7.38–7.44 (m, 4H), 7.46–7.50 (m, 2H), 7.81 (s, 1H), 7.91 (d, *J* = 7.0 Hz, 2H). ^13^C-NMR (100 MHz, CDCl_3_) δ ppm: 48.7 (CH), 49.4 (CH), 49.5 (CH), 52.8 (CH), 53.6 (OCH_3_), 55.4 (OCH_3_), 55.6 (OCH_3_), 59.1 (CH), 60.6 (CH), 80.1 (C), 111.1 (C), 114.3 (CH), 115.1 (CH), 119.3 (CH), 119.8 (CH), 120.5 (C), 123.7 (C), 123.8 (C), 126.4 (CH), 126.7 (CH), 126.9 (CH), 127.1 (CH), 127.4 (CH), 127.8 (CH), 128.0 (CH), 128.2 (CH), 128.3 (CH), 128.4 (CH), 128.5 (CH), 128.6 (CH), 129.1 (CH), 129.2 (CH), 132.3 (C), 132.6 (C), 139.6 (C), 139.7 (C), 150.5 (C), 152.7 (C), 159.3 (C), 160.1 (C), 170.2 (C), 172.7 (C), 173.9 (C), 175.6 (C), 176.3 (C). MS (EI, 70 eV) *m/z* (%): 955(M^+^), 751 (38), 521 (25), 233 (100), 203 (48). Elemental Analyses calcd. for C_57_H_45_N_7_O_8_.2H_2_O: C: 69.01, H: 4.98, N: 9.88. Found: C: 69.42, H: 5.18, N: 9.84.

*Methyl 2,5-bis(benzo[d][1,3]dioxol-5-ylmethyl)-7,9-bis(1,3-diphenyl-1H-pyrazol-4-yl)-1,3,4,6-tetra- oxododecahydro-1H-dipyrrolo[3,4-a:3',4'-f]pyrrolizine-3b-carboxylate* (**4j**). White Solid. Yield: 75%; m.p.: 241–243 °C. IR (KBr): ν 3647, 3062, 1744, 1700, 1599, 1503 cm^−1^. ^1^H-NMR (400 MHz, CDCl_3_) δ ppm: 3.34 (t, *J* = 8.3 Hz, 1H), 3.50 (t, *J* = 9.3 Hz, 1H), 3.92 (s, 3H), 4.20 (d, *J* = 13.8 Hz, 1H), 4.24–4.31 (m, 2H), 4.39 (dd, *J* = 13.8, 5.0 Hz, 2H), 4.49–4.54 (m, 1H), 4.62 (d, *J* = 8.3 Hz, 1H), 4.75 (d, *J* = 8.5 Hz, 1H), 5.75–5.85 (m, 2H), 5.95 (s, 2H), 6.34 (d, *J* = 7.8 Hz, 1H), 6.65–6.70 (m, 1H), 6.74 (d, *J* = 8.0 Hz, 1H), 6.85–6.92 (m, 3H), 6.98 (d, *J* = 7.3 Hz, 2H), 7.04–7.10 (m, 3H), 7.13–7.24 (m, 5H), 7.27–7.36 (m, 5H), 7.38–7.50 (m, 5H), 7.86 (d, *J* = 7.3 Hz, 2H). ^13^C-NMR (100 MHz, CDCl_3_) δ ppm: 42.1 (CH_2_), 42.9 (CH_2_), 48.2 (CH), 48.3 (CH), 50.0 (CH), 52.0 (CH), 53.6 (OCH_3_), 58.6 (CH), 60.0 (CH), 80.3 (C), 100.9 (CH_2_), 101.2 (CH_2_), 108.1 (CH), 108.4 (CH), 109.0 (CH), 109.5 (CH), 111.0 (C), 119.1 (C), 119.3 (CH), 120.0 (CH), 122.2 (CH), 122.7 (CH), 126.1 (CH), 126.6 (CH), 126.7 (CH), 127.6 (CH), 127.8 (CH), 127.9 (CH), 128.2 (C), 128.3 (CH), 128.4 (CH), 128.5 (CH), 128.6 (CH), 128.9 (CH), 129.2 (CH), 129.4 (C), 132.4 (C), 132.6 (C), 139.5 (C), 139.6 (C), 147.1 (C), 147.5 (C), 147.7 (C), 147.8 (C), 150.1 (C), 152.2 (C), 170.2 (C), 173.0 (C), 174.7 (C), 176.4 (C), 176.6 (C). MS (EI, 70 eV) *m/z* (%): 1011(M^+^), 780 (11), 547 (7), 257 (15), 233 (45), 135 (100). Elemental Analyses calcd. for C_59_H_45_N_7_O_10_.2H_2_O: C: 67.61, H: 4.71, N: 9.36. Found: C: 67.84, H: 4.39, N: 9.21.

### 3.3. Synthesis and Characterization Data for Pyrazolylpyrrolo[3,4-c]Pyrroles ***5a–f*** and ***6a–f***.

#### General Synthetic Procedure

To a 25.0 mL round bottom flask equipped with a magnetic stirring bar and a reflux condenser were added pyrazole-4-carboxaldehyde **1a**–**b** (0.2 mmol), *N*-substituted-maleimide **2a**–**e** (0.2 mmol), *N*-benzyl glycine ethyl ester **3b** (0.22 mmol) and toluene (8 mL). The mixture was heated under reflux until TLC showed the absence of the starting materials (6–10 h). After the reaction mixture was cooled down to room temperature, the solvent was removed under reduced pressure and the resulting crude product was purified by column chromatography on silica gel, using a mixture of dichloromethane/hexane (7:3) as eluent. In all cases the minor diastereomers were characterized by ^1^H-NMR spectroscopy after purification.

*Ethyl 2-benzyl-3-(3-methyl-1-phenyl-1H-pyrazol-4-yl)-4,6-dioxo-5-phenyloctahydropyrrolo[3,4-c]-pyrrole-1-carboxylate.* White Solid. Yield: 95%; m.p.: 172–174 °C. IR (KBr): ν 1717, 1598, 1502 cm^−1^. *Minor diastereomer* **5a**. ^1^H-NMR (400 MHz, CDCl_3_) δ ppm: 1.26 (t, *J* = 7.3 Hz, 3H), 2.38 (s, 3H), 3.34 (d, *J* = 13.8 Hz, 1H), 3.57 (dd, *J* = 9.7, 5.4 Hz, 1H), 3.86 (d, *J* = 4.8 Hz, 1H), 3.89 (d, *J* = 9.3 Hz, 1H), 4.20 (q, *J* = 7.3 Hz, 2H), 4.24 (d, *J* = 9.0 Hz, 1H), 4.79 (d, *J* = 5.5 Hz, 1H), 7.22–7.28 (m, 4H), 7.29–7.34 (m, 4H), 7.40–7.53 (m, 5H), 7.66 (d, *J* = 7.5 Hz, 2H), 7.92 (s, 1H). *Major diastereomer* **6a**^1^H-NMR (400 MHz, CDCl_3_) δ ppm: 1.32 (t, *J* = 7.2 Hz, 3H), 2.45 (s, 3H), 3.53 (d, *J* = 8.0 Hz, 1H), 3.65 (d, *J* = 14.0 Hz, 1H), 3.80–3.87 (m, 1H), 4.05 (d, *J* = 14.1 Hz, 1H), 4.17–4.34 (m, 2H), 4.39 (s, 1H), 4.94 (d, *J* = 9.5 Hz, 1H), 7.13 (d, *J* = 6.8 Hz, 2H), 7.19–7.30 (m, 4H), 7.31–7.42 (m, 7H), 7.52 (d, *J* = 7.8 Hz, 2H), 7.72 (s, 1H). ^13^C-NMR (100 MHz, CDCl_3_) δ ppm: 12.4 (CH_3_), 14.2 (CH_3_), 48.6 (CH), 48.7 (CH), 52.1 (CH_2_), 59.8 (CH), 60.9 (CH_2_), 62.9 (CH), 118.5 (CH), 125.5 (CH), 126.0 (CH), 127.3 (CH), 127.7 (CH), 128.2 (CH), 128.3 (CH), 128.6 (CH), 129.0 (CH), 129.2 (CH), 130.3 (C), 131.6 (C), 137.6 (C), 139.8 (C), 149.8 (C), 170.9 (C), 174.3 (C), 175.6 (C). MS (EI, 70 eV) *m/z* (%): 534 (M^+^, 15), 461 (65), 91 (100). Elemental Analyses calcd. for C_32_H_30_N_4_O_4_.2H_2_O: C: 67.35, H: 6.01, N: 9.82. Found: C: 67.45, H: 6.13, N: 9.66.

*Ethyl 2-benzyl-5-(4-chlorophenyl)-3-(3-methyl-1-phenyl-1H-pyrazol-4-yl)-4,6-dioxooctahydropyrrolo-[3,4-c]pyrrole-1-carboxylate.* Yellow Solid. Yield: 82%; m.p.: 69–71 °C. IR: ν 1782, 1738, 1598 cm^−1^. *Minor diastereomer* **5b**. ^1^H-NMR (400 MHz, CDCl_3_) δ ppm: 1.24 (t, *J* = 7.3 Hz, 3H), 2.36 (s, 3H), 3.31 (d, *J* = 13.8 Hz, 1H), 3.54 (dd, *J* = 9.5, 5.5 Hz, 1H), 3.77–3.85 (m, 3H), 4.22–4.30 (m, 2H), 4.75 (d, *J* = 5.3 Hz, 1H), 7.06 (d, *J* = 8.3 Hz, 2H), 7.18–7.23 (m, 2H), 7.29–7.34 (m, 3H), 7.37 (t, *J* = 7.8 Hz, 2H), 7.44 (d, *J* = 8.5 Hz, 1H), 7.49 (d, *J* = 8.0 Hz, 2H), 7.67 (d, *J* = 7.5 Hz, 2H), 7.91 (s, 1H). *Major diastereomer* **6b**. ^1^H-NMR (400 MHz, CDCl_3_) δ ppm: 1.30 (t, *J* = 7.2 Hz, 3H), 2.42 (s, 3H), 3.50 (d, *J* = 7.8 Hz, 1H), 3.63 (d, *J* = 14.3 Hz, 1H), 3.78–3.81 (m, 1H), 4.02 (d, *J* = 14.3 Hz, 1H), 4.15–4.22 (m, 2H), 4.35 (s, 1H), 4.91 (d, *J* = 9.5 Hz, 1H), 7.06 (d, *J* = 8.3 Hz, 2H), 7.18–7.23 (m, 3H), 7.29–7.34 (m, 3H), 7.37 (t, *J* = 7.8 Hz, 2H), 7.44 (d, *J* = 8.5 Hz, 1H), 7.49 (d, J = 8.0 Hz, 2H), 7.63–7.69 (m, 2H). ^13^C-NMR (100 MHz, CDCl_3_) δ ppm: 12.4 (CH_3_), 14.2 (CH_3_), 46.4 (CH), 48.7 (CH), 52.1 (CH_2_), 59.8 (CH), 60.9 (CH_2_), 62.9 (CH), 118.4 (CH), 119.6 (C), 126.1 (CH), 126.6 (CH), 127.3 (CH), 127.7 (CH), 128.6 (CH), 129.1 (CH), 129.2 (CH), 129.3 (CH), 130.1 (C), 133.9 (C), 137.5 (C), 139.7 (C), 170.8 (C), 170.8 (C), 174.1 (C), 175.3 (C). MS (EI, 70 eV) *m/z* (%): 568 (M^+^, 1), 497 (11), 496 (10), 495 (30), 91 (100). Elemental Analyses calcd. for C_32_H_29_ClN_4_O_5_.H_2_O: C: 65.47, H: 5.32, N: 9.54. Found: C: 65.28, H: 5.39, N: 9.76.

*Ethyl 2-benzyl-5-(4-methoxyphenyl)-3-(3-methyl-1-phenyl-1H-pyrazol-4-yl)-4,6-dioxooctahydro-pyrrolo[3,4-c]pyrrole-1-carboxylate.* White Solid. Yield: 96%; m.p.: 85–87 °C. IR: ν 1775, 1728, 1602 cm^−1^. *Minor diastereomer* **5c**. ^1^H-NMR (400 MHz, CDCl_3_) δ ppm: 1.26 (t, *J* = 7.2 Hz, 3H), 2.38 (s, 3H), 3.34 (d, *J* = 13.8 Hz, 1H), 3.55 (dd, *J* = 9.5, 5.3 Hz, 1H), 3.82–3.91 (m, 5H), 4.19 (q, *J* = 7.3 Hz, 2H), 4.23 (d, *J* = 8.8 Hz, 1H), 4.78 (d, *J* = 5.5 Hz, 1H), 7.00 (d, *J* = 9.0, 2H), 7.21–7.26 (m, 5H), 7.27–7.34 (m, 3H), 7.45 (t, *J* = 8.5, 7.3 Hz, 2H), 7.66 (dd, *J* = 8.7, 1.1 Hz, 2H), 7.91 (s, 1H). *Major diastereomer* **6c**. ^1^H-NMR (400 MHz, CDCl_3_) δ ppm: 1.32 (t, *J* = 7.2 Hz, 3H), 2.44 (s, 3H), 3.51 (d, *J* = 7.8 Hz, 1H), 3.65 (d, *J* = 14.3 Hz, 1H), 3.76 (s, 3H), 4.04 (d, *J* = 14.1 Hz, 1H), 4.16–4.32 (m, 3H), 4.38 (s, 1H), 4.92 (d, *J* = 9.5 Hz, 1H), 6.84 (d, *J* = 9.0 Hz, 2H), 7.04 (d, *J* = 8.8 Hz, 2H), 7.21–7.27 (m, 3H), 7.30–7.35 (m, 3H), 7.36–7.41 (m, 2H), 7.53 (d, *J* = 7.8 Hz, 2H), 7.70 (s, 1H). ^13^C-NMR (100 MHz, CDCl_3_) δ ppm: 12.4 (CH_3_), 14.2 (CH_3_), 46.4 (CH), 48.6 (CH), 52.0 (CH_2_), 55.3 (OCH_3_), 59.8 (CH), 60.9 (CH_2_), 62.9 (CH), 114.3 (CH), 118.5 (CH), 119.8 (C), 124.3 (C), 126 (CH), 126.8 (CH), 127.3 (CH), 127.7 (CH), 128.2 (CH), 128.6 (CH), 129.2 (CH), 137.7 (C), 139.8 (C), 149.8 (C), 159.1 (C), 171.0 (C), 174.5 (C), 175.8 (C). MS (EI, 70 eV) *m/z* (%): 564 (M^+^, 6), 493 (14), 492 (71), 491 (99), 473 (37), 270 (33), 91 (100). Elemental Analyses calcd. for C_33_H_32_N_4_O_5_.2H_2_O: C: 65.99, H: 6.04, N: 9.33. Found: C: 66.12, H: 6.24, N: 9.02.

*Ethyl 2-benzyl-3-(1,3-diphenyl-1H-pyrazol-4-yl)-4,6-dioxo-5-phenyloctahydropyrrolo[3,4-c]pyrrole-1-carboxylate.* White Solid. Yield: 75%; m.p.: 181–183 °C. IR (KBr): ν 1721, 1597, 1498 cm^−1^. *Minor diastereomer* **5d**. ^1^H-NMR (400 MHz, CDCl_3_) δ ppm: 1.27 (t, *J* = 7.1 Hz, 3H), 3.38 (d, *J* = 13.6 Hz, 1H), 3.72 (dd, *J* = 9.7, 5.6 Hz, 1H), 3.83 (d, *J* = 9.0 Hz, 1H), 3.89–3.93 (m, 1H), 4.28–4.32 (m, 3H), 5.05 (d, *J* = 5.5 Hz, 1H), 7.14–7.18 (m, 2H), 7.32–7.35 (m, 5H), 7.40–7.43 (m, 4H), 7.50–7.53 (m, 5H), 7.82–7.85 (m, 2H), 7.97–8.02 (m, 2H), 8.15 (s, 1H). *Major diastereomer* **6d**. ^1^H-NMR (400 MHz, CDCl_3_) δ ppm: 1.32 (t, *J* = 7.2 Hz, 3H), 3.58 (d, *J* = 7.8 Hz, 1H), 3.67 (d, *J* = 14.3 Hz, 1H), 3.92–3.98 (m, 1H), 4.06 (d, *J* = 14.1 Hz, 1H), 4.21–4.29 (m, 2H), 4.45 (s, 1H), 5.11 (d, *J* = 9.8 Hz, 1H), 7.22–7.26 (m, 3H), 7.30 (dd, *J* = 7.4, 1.9 Hz, 2H), 7.32–7.35 (m, 1H), 7.36–7.40 (m, 4H), 7.43–7.48 (m, 2H), 7.49–7.52 (m, 2H), 7.55–7.60 (m, 2H), 7.65 (d, *J* = 7.5 Hz, 2H), 7.86–7.90 (m, 2H), 7.92 (s, 1H). ^13^C-NMR (100 MHz, CDCl_3_) δ ppm: 14.3 (CH_3_), 49.0 (CH), 49.5 (CH), 52.3 (CH_2_), 60.2 (CH), 61.1 (CH_2_), 63.1 (CH), 118.9 (CH), 119.0 (CH), 119.1 (C), 125.6 (CH), 126.1 (CH), 126.6 (CH), 126,7 (CH), 127.4 (CH), 128.4 (CH), 128.5 (CH), 128.6 (CH), 128.7 (CH), 129.2 (CH), 129.4 (CH), 131.8 (C), 133.1 (C), 137.8 (C), 139.9 (C), 153.3 (C), 171.0 (C), 174.6 (C), 175.7 (C). MS (EI, 70 eV) *m/z* (%): 596 (M^+^, 4), 523 (100), 505 (32). Elemental Analyses calcd. for C_37_H_32_N_4_O_4_.H_2_O: C: 72.30, H: 5.58, N: 9.11. Found: C: 72.68, H: 5.28, N: 9.05.

*Ethyl 2-benzyl-5-(4-chlorophenyl)-3-(1,3-diphenyl-1H-pyrazol-4-yl)-4,6-dioxooctahydropyrrolo[3,4-c]pyrrole-1-carboxylate.* White Solid. Yield: 72%; m.p.: 108–110 °C. IR (KBr): ν 1719, 1599, 1496 cm^−1^. *Minor diastereomer* **5e**. ^1^H-NMR (400 MHz, CDCl_3_) δ ppm: 1.23 (t, *J* = 7.0 Hz, 3H), 3.33 (d, *J* = 13.6 Hz, 1H), 3.68 (dd, *J* = 9.7, 5.6 Hz, 1H), 3.75–3.83 (m, 1H), 3.86–3.89 (m, 1H), 3.92 (d, *J* = 8.0 Hz, 1H), 4.15–4.24 (m, 2H) 4.97 (d, *J* = 5.5 Hz, 1H), 7.09–7.13 (m, 2H), 7.22–7.26 (m, 3H), 7.32–7.36 (m, 2H), 7.41 (br. s., 3H), 7.50 (d, *J* = 3.3 Hz, 2H), 7.52–7.55 (m, 3H), 7.78 (br. s., 3H), 7.94 (d, *J* = 6.8 Hz, 1H), 8.10 (s, 1H). *Major diastereomer* **6e**. ^1^H-NMR (400 MHz, CDCl_3_) δ ppm: 1.28 (t, *J* = 7.2 Hz, 3H), 3.53 (d, *J* = 7.8 Hz, 1H), 3.62 (d, *J* = 14.1 Hz, 1H), 3.88–3.95 (m, 1H), 4.00 (d, *J* = 14.3 Hz, 1H), 4.12–4.21 (m, 2H), 4.39 (s, 1H), 5.05 (d, *J* = 9.8 Hz, 1H), 7.15–7.20 (m, 3H), 7.20–7.26 (m, 2H), 7.29–7.32 (m, 4H), 7.39–7.48 (m, 4H), 7.49–7.55 (m, 2H), 7.58 (d, *J* = 7.8 Hz, 2H), 7.76–7.85 (m, 3H). ^13^C-NMR (100 MHz, CDCl_3_) δ ppm: 14.2 (CH_3_), 48.9 (CH), 49.4 (CH), 52.2 (CH_2_), 60.1 (CH), 61.0 (CH_2_), 63.0 (CH), 118.8 (CH), 125.8 (C), 126.7 (CH), 127.4 (CH), 127.8 (CH), 128.3 (CH), 128.4 (CH), 128.5 (CH), 128.5 (CH), 128.7 (CH), 129.1 (CH), 129.3 (CH), 129.4 (CH), 130.2 (C), 132.9 (C), 134.1 (C), 137.6 (C), 139.7 (C), 153.2 (C), 170.8 (C), 174.2 (C), 175.4 (C). MS (EI, 70 eV) *m/z* (%): 630 (M^+^, 2), 557 (49), 554 (43), 553 (100), 535 (45). Elemental Analyses calcd. for C_37_H_31_ClN_4_O_4_: C: 70.41, H: 4.95, N: 8.88. Found: C: 70.72, H: 5.04, N: 8.54.

*Ethyl 2-benzyl-3-(1,3-diphenyl-1H-pyrazol-4-yl)-5-(4-methoxyphenyl)-4,6-dioxooctahydropyrrolo[3,4-c]pyrrole-1-carboxylate.* Yellow Solid. Yield: 90%; m.p.: 78–80 °C. IR: ν 1712, 1600, 1548 cm^−1^. *Minor diastereomer* **5f**. ^1^H-NMR (400 MHz, CDCl_3_) δ ppm: 1.26 (t, *J* = 7.0 Hz, 3H), 3.37 (d, *J* = 13.6 Hz, 1H), 3.71 (d, *J* = 5.5 Hz, 1H), 3.82–3.86 (m, 4H), 3.88–3.91 (m, 1H), 4.21–4.31 (m, 3H), 5.04 (d, *J* = 5.5 Hz, 1H), 7.01 (d, *J* = 9.0, 2H), 7.27–7.32 (m, 6H), 7.49–7.56 (m, 7H), 7.84 (d, *J* = 7.8, 2H), 8.00 (d, *J* = 7.0, 2H), 8.15 (s, 1H). *Major diastereomer* **6f**. ^1^H-NMR (400 MHz, CDCl_3_) δ ppm: 1.31 (t, *J* = 7.2 Hz, 3H), 3.55 (d, *J* = 8.0 Hz, 1H), 3.66 (d, *J* = 14.3 Hz, 1H), 3.80 (s, 3H), 3.93 (dd, *J* = 9.5 Hz, 8.0 Hz 1H), 4.05 (d, *J* = 14.1 Hz, 1H), 4.15–4.28 (m, 2H), 4.44 (s, 1H), 5.10 (d, *J* = 9.5 Hz, 1H), 6.89 (d, *J* = 9.0 Hz, 2H), 7.16 (d, *J* = 8.8 Hz, 2H), 7.21–7.27 (m, 2H), 7.28–7.32 (m, 2H), 7.32–7.38 (m, 2H), 7.42–7.52 (m, 3H), 7.57 (t, *J* = 7.4 Hz, 2H), 7.66 (d, *J* = 7.5 Hz, 2H), 7.88 (d, *J* = 7.0 Hz, 2H), 7.91 (s, 1H). ^13^C-NMR (100 MHz, CDCl_3_) δ ppm: 14.1 (CH_3_), 48.7 (CH), 49.3 (CH), 52.1 (CH_2_), 55.3 (OCH_3_), 60.0 (CH), 60.9 (CH_2_), 63.0 (CH), 114.2 (CH), 118.7 (CH), 119.8 (C), 124.3 (C), 126.4 (CH), 126.7 (CH), 127.2 (CH), 127.7 (CH), 128.1 (CH), 128.3 (CH), 128.4 (CH), 128.5 (CH), 129.0 (CH), 129.3 (CH), 132.9 (C), 137.7 (C), 139.7 (C), 153.1 (C), 159.1 (C), 170.8 (C), 174.6 (C), 175.8 (C). MS (EI, 70 eV) *m/z* (%): 626 (M^+^, 4), 554 (39), 553 (100), 535 (42). Elemental analyses calcd. for C_38_H_34_N_4_O_5_.H_2_O: C: 70.79, H: 5.63, N: 8.69. Found: C: 70.56, H: 5.81, N: 8.51.

## 4. Conclusions

We described here a practical synthesis of pyrazoylpyrrolizines **4** and pyrazolylpyrrolidines derivatives **5** and **6** from pyrazolyl-carboxaldehydes, glycine derivates and maleimides by a three-component catalyst free domino process involving both the formation of a 1,3-dipolar species and 1,3-cycloaddition reaction to afford the desired products in good yields and with good atom economy. This high-throughput methodology provides an easy execution, rapid access and good diastereoselectivity. When the *N*-benzyl glycine ethyl ester was used two diastereomers **5** and **6** were obtained, with the diastereomers **6a-f** being favored by the minor repulsive interaction between the carbonyl group on 1-C and 6-C=O carbon due to their *trans* configuration.
